# First report on identification and genetic characterization of Getah virus in wild boar in China

**DOI:** 10.3389/fmicb.2025.1583023

**Published:** 2025-03-24

**Authors:** Jian Huang, Deping Song, Jiazhen Wei, Baobao Xie, Qun Yang, Jun Xu, Fanfan Zhang

**Affiliations:** ^1^Key Laboratory of Agro-product Quality and Safety of Jiangxi Province, Institute for Quality & Safety and Standards of Agricultura Sciences, Jiangxi Academy of Agricultural Sciences, Nanchang, Jiangxi, China; ^2^College of Animal Science and Technology, Jiangxi Agricultural University, Nanchang, Jiangxi, China; ^3^Department of Animal Science, Jiangxi Biological Vocational College, Nanchang, Jiangxi, China; ^4^Dabeinong Technology Co., Ltd. of Jiangxi, Nanchang, Jiangxi, China; ^5^Key Laboratory of Green and Healthy Breeding of Livestock and Poultry of Jiangxi Province, Institute of Animal Husbandry and Veterinary Medicine, Jiangxi Academy of Agricultural Sciences, Nanchang, Jiangxi, China

**Keywords:** Getah virus, epidemiology, genomic sequencing, phylogenetic analysis, wild boar

## Abstract

Getah virus (GETV) is a mosquito-borne *Alphavirus* that causes fever, rash and oedema in horses, fever, anorexia and neurological symptoms as well as piglet diarrhea deaths and reproductive disorders in sows in blue foxes, and the endemic areas are expanding progressively, especially in Asia and Oceania. In order to study the current epidemiological status of GETV in wild boar herds and its genetic and evolutionary patterns, we conducted a survey and genomic analyses of GETV in pig herds. The results showed that 74.02% (57/77) of the samples tested (*n* = 77) were positive for GETV, with the highest positivity rate of 100% in visceral tissue samples from diarrheic piglets and aborted fetuses, and also for the first time, the presence of GETV was detected in milk and semen. The complete genome of the representative GETV strain JX2024 was determined, and genome wide and E2 gene evolution tree results showed that the representative strain belonged to subtype GIII. Multiple comparison analysis showed that the E2 gene of JX2024 differed at the amino acid level by 96.4% to 99.5% compared with the reference GETV strain, and that the mutation of arginine to lysine at position 253 was the key amino acid site for the enhanced virulence of GETV. This analysis bridges the gap between the molecular epidemiological data and the genetic variation of GETV in wild boar and provides a basis for further understanding of the spread of GETV in China.

## 1 Introduction

Getah virus (GETV) is a member of the genus *Alphavirus* in the family *Togaviridae*, and is an arbovirus (Li et al., 2022; Shi et al., 2022b). GETV is widely distributed around the world, and has been reported in many countries, including Australia, the Philippines, India, Japan, Korea, China, and Russia, after it was isolated from the Culex gelidus, which survives in the rubber forests of Malaysia, for the first time in 1955 (Brown and Timoney, 1998; Feng et al., 2012; Hasan et al., 2025; Li et al., 2017; Rattanatumhi et al., 2022; Rivera et al., 2024; Sam et al., 2022; Yuen et al., 2022). The virus has a wide spectrum of infection, affecting horses, pigs, cattle, and blue foxes, among others (Liu et al., 2019; Sentsui and Kono, 1985; Shi et al., 2019; Xing et al., 2020). Infected horses exhibit symptoms such as fever, rash, hind limb oedema, and enlarged lymph nodes, while infected pigs primarily manifest fever, arthritis, and the death of neonatal piglets, as well as reproductive disorders in gestating sows, resulting in economic losses to the farming industry (Lu et al., 2019; Xing et al., 2020). Swine are considered to be the primary amplifying host of GETV in nature, and virus-specific antibodies have been detected in the sera of both febrile patients and healthy individuals in endemic areas, suggesting that GETV can infect humans, which is of great public health significance (Li et al., 2022, 1992).

The GETV virus particles are planet-shaped, with a fusiform membrane and fibrils, with a diameter of about 70 nm. the genome is a single-stranded, positive-stranded RNA, with a total length of about 11–12 knt, with a methylated cap structure at the 5′ end of the genome, and a Poly (A) tail at the 3′ end of the genome (Chu et al., 2024; Li et al., 2022; Matsumura et al., 2024). The genome contains two open reading frames (ORFs) encoding four non-structural proteins (nsP1, nsP2, nsP3 and nsP4) and five structural proteins (CP, E3, E2, 6K, and E1). There is also a sub-genomic promoter 26S between the non-structural and structural proteins, and the complex recognizes the 26S promoter to synthesize 26SmRNA, which initiates transcription of the structural protein genes. E2 protein is an important component of GETV. Together with E1, E2 constitutes the vesicle membrane of the virus, which is capable of entering the host cell through endocytosis and binding to the virus by attachment factors on the cell membrane. In addition, the E2 protein is also a viral antigen recognition and neutralization site, which can elicit an immune response from the host.

In recent years, swine morbidity caused by GETV infection has been repeatedly reported. In 2017, an outbreak of GETV in a pig farm in Hunan Province resulted in the deaths of approximately 200 newborn piglets as well as more than 150 gestating sows giving birth to stillborn or mummified fetuses (Yang et al., 2018); this was followed by GETV outbreaks in pig factories in Guangdong Province and other places (Pan et al., 2023; Xing et al., 2020). There have been studies on GETV in China, but these have been limited to molecular epidemiology and to domestic pigs. In this study, we investigated the prevalence of infection in diarrheic wild boars. Elucidating the relationship of genetic characteristics and evolution to GETV in other countries/regions, we identified and analyzed representative GETVs in full-length genome sequence wild boars.

## 2 Materials and methods

### 2.1 Ethics statement

All samples were collected on commercial wild boar farms by veterinarians during routine diagnostic sampling after permission from the farm owner. No specific permits from an animal ethics committee were required.

### 2.2 Clinical signs and diagnostics

In October 2024, a medium-sized wild boar farm in Jiangxi, China, with a total population of 550 wild boars, observed symptoms such as diarrhea, fever, skin edema, and arthritis in suckling piglets. The farm maintained a strict vaccination protocol for common pig diseases but had not vaccinated against GETV, as no commercial vaccine was available at the time. Post-mortem examinations revealed hemorrhagic and enlarged inguinal lymph nodes, small intestines filled with abundant yellow content accompanied by hemorrhagic and enlarged mesenteric lymph nodes, pinpoint hemorrhages in the kidneys, subcutaneous edema in the neck and chest regions with partial jaundice, and an incidence rate of 75% among affected piglets. Subsequently, sows exhibited an increased rate of estrus return, abortions, stillbirths, or the birth of weak piglets, while boar sperm quality declined. The overall incidence rate among suckling piglets was 75%, with a mortality rate exceeding 90%. A total of 30 diarrhea samples, 30 sera from diseased pigs, five visceral tissue samples (lymph nodes and lungs), two aborted fetuses, five sow milk samples, and five boar semen samples were collected and sent to our laboratory for pathogen identification. Initially, standard detection methods were employed to screen for common diarrhea viruses, reproductive disorder viruses, and bacterial pathogens, including porcine epidemic diarrhea virus (PEDV), transmissible gastroenteritis virus (TGEV), porcine rotavirus (PoRV), porcine deltacoronavirus (PDCoV), swine acute diarrhea syndrome coronavirus (SADS-CoV), porcine circovirus 2 (PCV-2), Porcine circovirus 3 (PCV-3), African swine fever virus (ASFV), classical swine fever virus (CSFV), porcine reproductive and respiratory syndrome virus (PRRSV), Japanese encephalitis virus (JEV), Porcine Parvovirus (PPV), as well as pathogenic *Escherichia coli* (*E. coli*), and *Salmonella* spp. were investigated.

### 2.3 Detection of GETV and other related pathogens

Total RNAs/DNAs were extracted using CHINREAL Mini Viral RNA/DNA Extraction Kit (CHINREAL, Shenzhen, China) was used according to the manufacturer's instructions on the Purifier 32 automated extraction system (CHINREAL, Shenzhen, China) and then were stored at −80°C until used. To investigate whether GETV was associated within these cases, a pair of primers (GETV-F: 5′-CCAACTCAAACCTTTTACGGAC-3′, and GETV-R: 5′- TTTACCTGCGCCTGTCGGGA-3′ with a predicted product size of 636 bp) for a polymerase chain reaction (RT-PCR) was initially designed based on the Cap gene of HuN1 (GenBank accession no. MF741771), and then a RT-PCR assay was established with the designed primers. The first-strand cDNA synthesis was performed at 25°C for 5 min, 50°C for 45 min and then 85°C for 2 min to inactivate the HiScript II 1st Strand cDNA Synthesis Kit (Vazyme, Nanjing, China) and followed by 4°C for the consecutive treatment.

For the amplification of the fragments, 2×Es Taq MasterMix (with Dye) (CoWin Bio., China) was used. The amplification conditions comprised an initial denaturation at 95°C for 5 min, followed by 32 cycles of denaturation at 95°C for 30 seconds, annealing at 54°C for 40 seconds, and extension at 72°C for 45 seconds. A final extension step at 72°C for 5 min was also incorporated. The expected PCR products were then purified, cloned, and sequenced following standard protocols. The previously established PCR protocols were used to test other viruses, PEDV, PDCoV, TGEV, SADS-CoV, PRoV, PCV2, PCV3, ASFV, PRRSV, JEV, and PPI (primer sequences present in Supplementary Table S1) (Zhang et al., 2024). In addition, common enteropathogenic germs, including pathogenic Escherichia coli, and Salmonella were also tested via bacterial isolation and identification with the standard protocols.

### 2.4 Complete genome amplification, sequencing and analysis of GETV

To amplify the full genome sequence of field strains of GETV, primers were developed using the GETV HuN1 strain (GenBank accession number MF741771) as a reference (see Table 1 for primer sequences). Viral DNA/RNA was isolated using a previously described method. Fragments were amplified using 2 × Phanta Max Master Mix (Dye Plus) (Vazyme, Nanjing, China), under the following conditions: an initial denaturation at 95°C for 3 min, followed by 30 cycles of 95°C for 15 seconds, 55°C for 15 seconds, and 72°C for 1 min, with a final extension at 72°C for 5 min. For determining the terminal sequences of GETV, rapid amplification of cDNA ends (RACE) was performed at both the 5′ and 3′ ends using the 5′/3′ SMARTer RACE kit (Clontech, Beijing, China), according to the manufacturer's guidelines. Positive PCR products were then purified by gel electrophoresis and cloned into pMD 18-T vectors (TaKaRa, Japan). Three to five positive clones from each amplified fragment were sent to a commercial sequencing company (Sangon Biotech, Shanghai, China) for bidirectional Sanger sequencing. The complete genome sequences were assembled and annotated utilizing SeqMan in DNAStar Lasergene version 7.10 (DNAStar, Inc., Madison, WI, USA). Phylogenetic studies of GETV were carried out based on the complete genome sequences and the deduced amino acid (aa) sequences of the E2 protein of GETV, employing the neighbor-joining method with 1,000 bootstrap replicates in Molecular Evolutionary Genetics Analysis (MEGA) software version 6.0.2.

**Table 1:** Oligonucleotide primers used for amplification of the complete genome of Getah virus by reverse transcription polymerase chain reaction.

**Name**	**Sequence (5^′^-3^′^)**	**Position^a^**	**Product size (bp)**
GETV 1F	ATGGCGGACGTGTGACATCAC	0–21	994
GETV 1R	GGGTAACTGCGTAATCGACTGTT	972–994	
GETV 2F	CGAGAGCCGAAAGCTGCTGC	822–841	950
GETV 2R	TTTGGGGGGAAAGGATCAAGT	1,751–1,771	
GETV 3F	GCGGCAGAAGAAGAAGAGAAGGA	1,544–1,565	905
GETV 3R	CACGCGAACGCCTCATCGACGTA	2,428–2,450	
GETV 4F	GCCAGTGGAAAGAAGGAGAACTG	2,299–2,321	868
GETV 4R	TCATACGTATTCCGGCCGTCTC	3,142–3,163	
GETV 5F	GTGACCCGTGGATAAAGGTGT	2,963–2,983	936
GETV 5R	GCGAGATGGTGGTGACAGCCC	3,878–3,898	
GETV 6F	ACTGAGTACCGGCAACACCACTA	3,739–3,761	1,093
GETV 6R	AATCGGCGTCCTCTACTGGGCAT	4,809–4,831	
GETV 7F	GGCGGTCGACATGGCTGAAAT	4,701–4,721	904
GETV 7R	ACCGAACTGTATTCCTGTGCT	5,584–5,604	
GETV 8F	ACCTAGACATCCAATTCGGTGAC	5,522–5,544	1,025
GETV 8R	GGTGCCTGGTGTAACTTTCACATC	6,523–6,546	
GETV 9F	GTGCCAATGGACCGCTTCGTGAT	6,487–6,509	997
GETV 9R	TCTATTTAGGACCGCCGTACAGA	7,461–7,483	
GETV 10F	AGGCTGTTCAAACTCGGGAA	7,228–7,247	1,010
GETV 10R	TCCCAGGACAATGGCCACCAC	8,217–8,237	
GETV 11F	ATCCCGACAGGCGCAGGTAAACC	8,154–8,176	877
GETV 11R	GCATGTCGATCTCTTCCTCCGT	9,009–9,030	
GETV 12F	GCCACTTCATCGTGGCCTACTG	8,815–8,836	1,035
GETV 12R	CCAGCCAAAACAGGGTTTGATTC	9,827–9,849	
GETV 13F	TGGGCCCAGCTGACAACTGAAG	9,534–9,555	1,467
GETV 13R	AGCTGCCTCCTGTATGGTGGCTA	10,978–11,000	
GETV 5′RACE	CAGTCCCCGATGCTTTCGCCAG	373–394	/
GETV 3′RACE	GGCGGTCTGCACGCACTCATCG	10,880–10,901	/

a Nucleotide position is numbered based on Avian encephalomyelitis virus strain HuN1 (MF741771).

## 3 Results

### 3.1 Prevalence of GETV and co-infections in wild boar

Among the 77 samples tested, 57 (74.02%) were positive for GETV, of which 20 (20/30, 66.67%) were diarrhea fecal samples, 25 (25/30, 83.33%) were serum samples, 5 (5/5, 100.00%) were visceral tissue samples from diarrhea piglets, 2 (2/2, 100.00%) were samples from aborted fetuses, 3 (3/5, 60.00%) were milk samples and 2 (2/5, 40.00%) were aborted fetal samples. In addition, we tested for co-infections in these samples. Of the 77 samples, 7 (7/77, 9.09%) were co-infected with GETV and PRoV; 1 (1/77, 1.30%) sample was co-infected with GETV and PRV. Forty nine of these samples were found to be positive only for GETV but negative for other diarrhea-associated and reproductive disorder-associated viruses, including PEDV, PDCoV, TGEV, SADS-CoV, PCV2, PCV3, ASFV, PRRSV, JEV, and PPI and any other known bacterial pathogens including pathogenic *E. coli, Salmonella*, etc.

### 3.2 Complete genome sequencing and genetic analysis of GETV

To characterize the genetic features of GETV, the complete genome sequence of one representative GETV strain, designated JX2024, was determined and analyzed. The genome length of the GETV strains isolated from dead fetuses was 11,689 bp (excluding the polyA tail), consistent with the genome size of other reported GETV strains (the sequence was deposited into the GenBank under the accession number of PV089541). Like other GETV genomes in GenBank, the JX2024 genome consists of a 5′ UTR, a 3′ UTR, and two open reading frames in between, with 78 nucleotides at the beginning of the 5′ end and the last 403 nucleotides at the 3′ end in the non-coding region, and 7,401 nucleotides following the non-coding region at the 5′ end, coding for the four structural proteins (NSP1, NSP2, NSP3, and NSP4), and 3,759 nucleotides preceding the non-coding region at the 3′ end coding for five structural proteins (C, E3, NSP3, and NSP4). NSP1, NSP2, NSP3, and NSP4), 3,759 nucleotides before the non-coding region at the 3′ end encoded five structural proteins (C, E3, E2, 6K, and E1), and 44 nucleotides were non-coding junctions between the two open reading frames.

**Figure 1:**
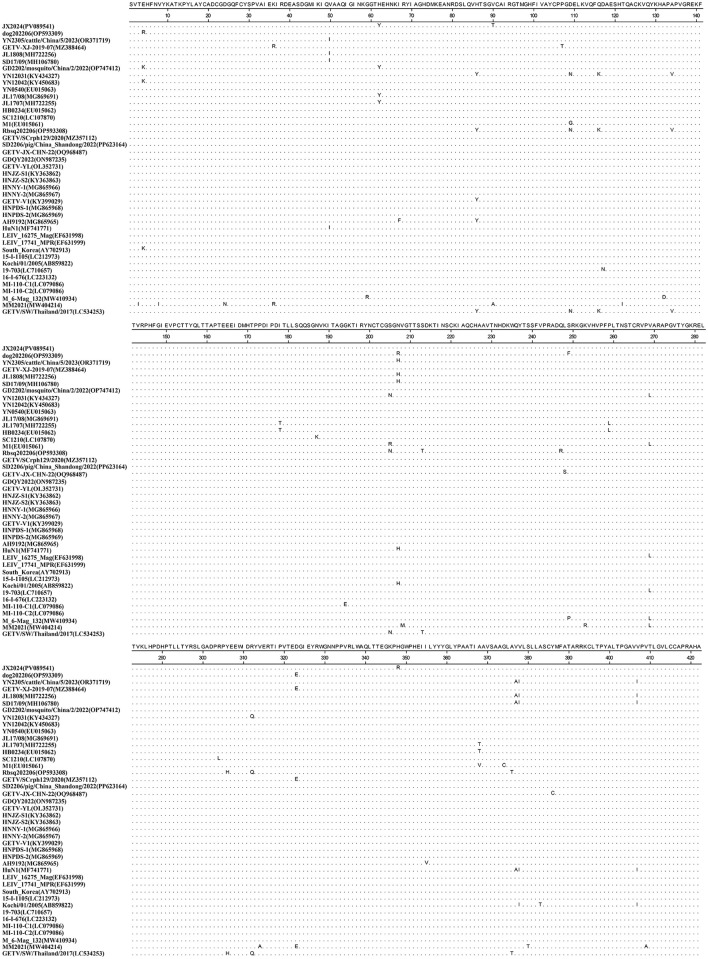
Alignment analysis of the amino acid sequences of the E2 genes between the identified GETV strain JX2024 and reference GETV strains.

**Figure 2:**
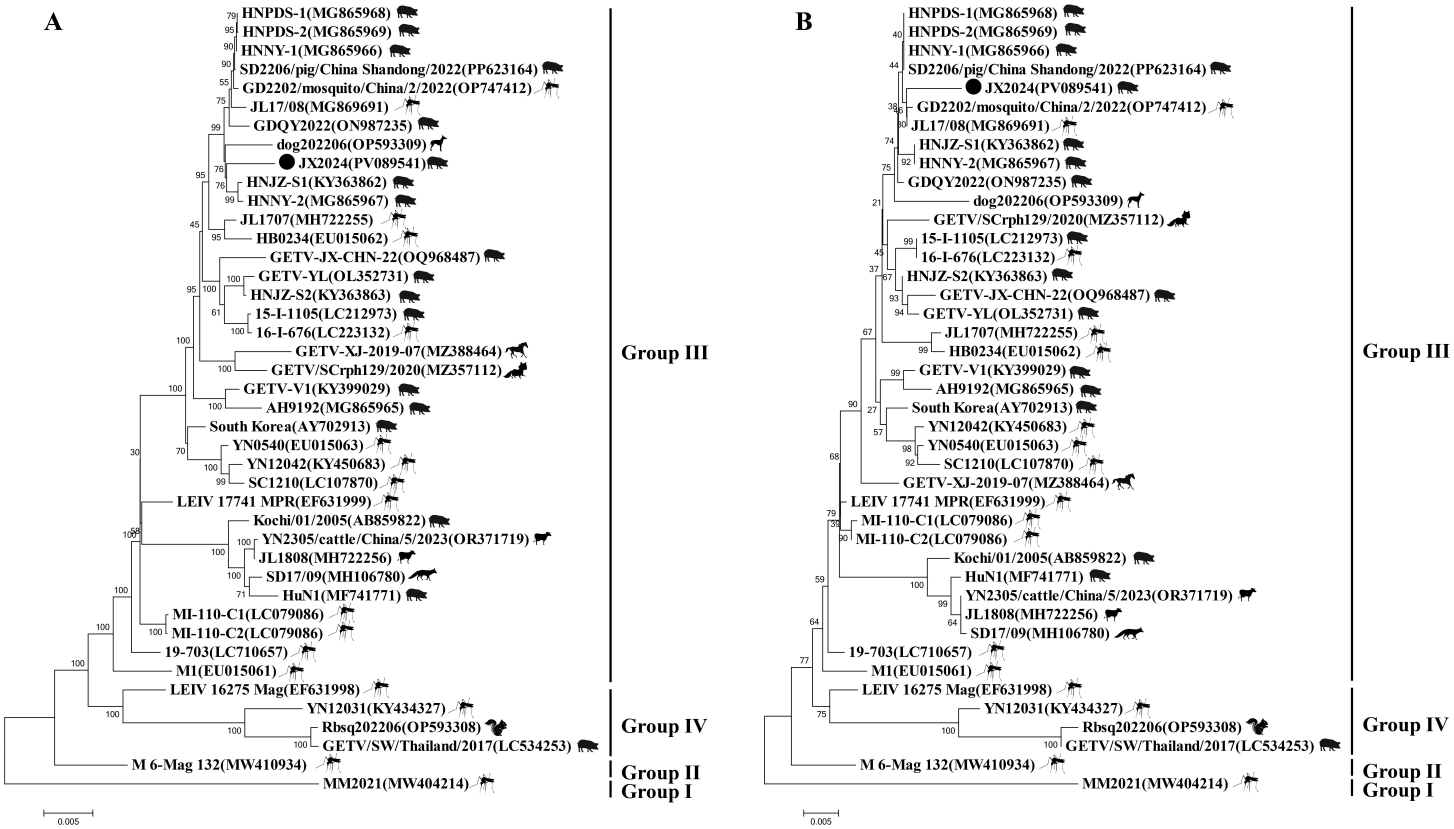
Phylogenetic trees generated based on the complete genome sequence of GETV **(A)** and E2 protein sequences of GETV around the world **(B)**. Bootstrap values were calculated with 1,000 replicates. A bar of 0.002 Indicates nucleotide or amino acid substitutions persite. “•” indicates the strain identified in this study.

Similarity analysis of the whole genome nucleotide sequence of GETV showed that the nucleotide similarity between strain JX2024 and 41 strains of Getavirus (Table 2) isolated from different geographical regions and hosts from 1955–2023 ranged from 94.4% to 99.4%, of which strain JX2024 was similar to the Chinese swine-origin strains SD2206/pig/China Shandong/2022 (PP623164), HNNY-1 (MG865966), HNNY-2 (MG865967), and HNPDS-1 (MG865968), with the highest similarity of 99.4%; and with Malaysian mosquito-origin strain MM2021 (AF339484), with the lowest similarity of 94.4% (Supplementary Table S2). The E2 protein is the main antigen of GETV, which is involved in binding host cell receptors and is the main protein that infects host cells, causes disease and triggers the host immune response. The nucleotide and amino acid sequences of the *GETV E2* gene were analyzed for similarity, and the results showed that the nucleotide and amino acid similarities between JX2024 and the reference strain of GETV ranged from 93.4% to 99.2%, and the amino acid similarities ranged from 96.4% to 99.5%, with the highest nucleotide and amino acid similarities between JX2024 and the Chinese mosquito strain JL17/08 (MG869691). The nucleotide and amino acid similarities between JX2024 and the Chinese mosquito strain JL17/08 (MG869691) were the highest, 99.2% and 99.5%, respectively, with the lowest nucleotide and amino acid similarities between JX2024 and the Chinese mosquito strain MM2021 (AF339484), 93.4% and 96.4%, respectively (Supplementary Table S3). Further analyses showed that the JX2024 strain had two unique amino acid mutation sites V90T and H348R in the E2 protein with other reference strains, and 15 amino acid differences with the protein of Malaysian mosquito strain MM2021 (AF339484) (Figure 1). GETV strains can be classified into four genotypes according to their gene sequences, and in this study, the GETV full gene, the E2 protein and the E2 protein of the Malaysian mosquito strain were established. The phylogenetic tree of the nucleotide sequences of the whole *GETV* gene and the *E2* gene was established in this study, and the results showed that the JX2024 strain belonged to the GIII group (Figure 2). The analysis of the genetic evolution of the whole genome of GETV showed that the JX2024 strain belonged to the GIII group with the dog isolate dog202206 (OP593309), the swine isolate HNJZ-S1 (KY363862), the pig isolate HNNY-2 (MG865969), and the swine isolate HNNY-2 (MG865969), which were all of GETV. (MG865967) were genetically evolved closer to each other, whereas they were most distantly related to the Malaysian mosquito isolate MM2021 (AF339484) and the Japanese mosquito isolate M 6-Mag 132 (MW410934). Analysis of the genetic evolution of the GETV E2 showed that the JX2024 strain was genetically evolved to be related to the mosquito isolate GD2202/mosquito/China/2/2022 (OP747412). The analysis of genetic evolution of GETV E2 showed that strain JX2024 was genetically evolved closer to strains GD2202/mosquito/China and JL17/08 (MG869691), while it was the most distantly related to Malaysian mosquito-origin isolate MM2021 (AF339484) and Japanese mosquito-origin isolate M 6-Mag 132 (MW410934).

**Table 2:** The sequence information used in this article.

**Isolate name**	**Time**	**Location**	**Host**	**Accession number**
▴JX2024	Oct-2024	China (Jiangxi)	Wild boar	
dog202206	Jun-2022	China (Heilongjiang)	Dog	OP593309
YN2305/cattle/China/5/2023	Mar-2023	China	Cattle	OR371719
GETV-XJ-2019-07	2019	China	Horse	MZ388464
JL1808	Aug-2018	China (Jilin)	Cattle	MH722256
SD17/09	Sep-2017	China (Shandong)	Fox	MH106780
GD2202/mosquito/China/2/2022	Feb-2022	China	Culex sp. (mosquito)	OP747412
YN12031	Jul-2012	China (Yunnan)	Armigeres subalbatus	KY434327
YN12042	Jul-2012	China (Yunnan)	Culex tritaeniorhynchus	KY450683
YN0540	2005	China (Yunnan)	Armigeres subalbatus	EU015063
JL17/08	Aug-2017	China (Jilin)	Mosquito	MG869691
JL1707	Aug-2017	China (Jilin)	Mosquito	MH722255
HB0234	2008	China (Hebei)	Culex tritaeniorhynchus Giles	EU015062
SC1210	Aug-2012	China (sichuan)	Armigeres subalbatus	LC107870
M1	2008	China (Hainan)	Culex sp.	EU015061
Rbsq202206	Jun-2022	China (Fujian)	Red-bellied tree squirrel	OP593308
GETV/SCrph129/2020	2020	China (Sichuan)	Ailurus fulgens	MZ357112
SD2206/pig/China Shandong/2022	Jun-2022	China (Shangdong)	Pig	PP623164
GETV-JX-CHN-22	2022	China (Jiangxi)	Swine	OQ968487
GDQY2022	Jun-2022	China (Guangdong)	Domestic pig	ON987235
GETV-YL	Sep-2021	China	Pig	OL352731
HNJZ-S1	Jan-2011	China	Pig	KY363862
HNJZ-S2	Aug-2015	China	Pig	KY363863
HNNY-1	Aug-2016	China	Pig	MG865966
HNNY-2	Sep-2016	China	Pig	MG865967
GETV-V1	Jul-2016	China	Pig	KY399029
HNPDS-1	Feb-2017	China	Pig	MG865968
HNPDS-2	Feb-2017	China	Pig	MG865969
AH9192	Oct-2017	China	Pig	MG865965
HuN1	Jul-2017	China	Pig	MF741771
LEIV 16275 Mag	2007	Russia	Aedes sp.	EF631998
LEIV 17741 MPR	2007	Mongolia	Culex sp.	EF631999
South Korea	2004	South Korea	Swine	AY702913
15-I-1105	2015	Japan	Sus scrofa domesticus	LC212973
Kochi/01/2005	2005	Japan	Sus scrofa	AB859822
19-703	Oct-2019	Japan	Equus caballus	LC710657
16-I-676	2016	Japan	Equus caballus	LC223132
MI-110-C1	1978	Japan	Equus caballus	LC079086
MI-110-C2	1978	Japan	Equus caballus	LC079087
M 6-Mag 132	Jul-1956	Japan	mosquito	MW410934
MM 2021	1955	Malaysia	Mosquito	AF339484
GETV/SW/Thailand/2017	May-2017	Thailand	Pig	LC534253

▴ Obtained in this research.

### 3.3 Clinical signs, gross lesions, and histopathological changes

GETV was detected in all tissues collected from diarrheic piglets and in all tissues collected from aborted fetuses, except brain tissue. These results suggest that GETV can be transmitted via the placenta and may infect newborn piglets. Gastrointestinal tissues from infected piglets were collected and analyzed. Upon gross observation, the stomach and small intestine of the infected piglets contained undigested celiac milk and fermented to produce large amounts of gas; the small intestine had a thin, transparent wall and was filled with yellowish-green, watery contents with a fishy odor; the mesentery was congested and severely swollen; the lungs were slightly swollen, with spots of bruising on the right lobe of the lungs; there were hemorrhagic spots on the kidneys; and there were no obvious lesions in other tissues and organs.

**Figure 3:**
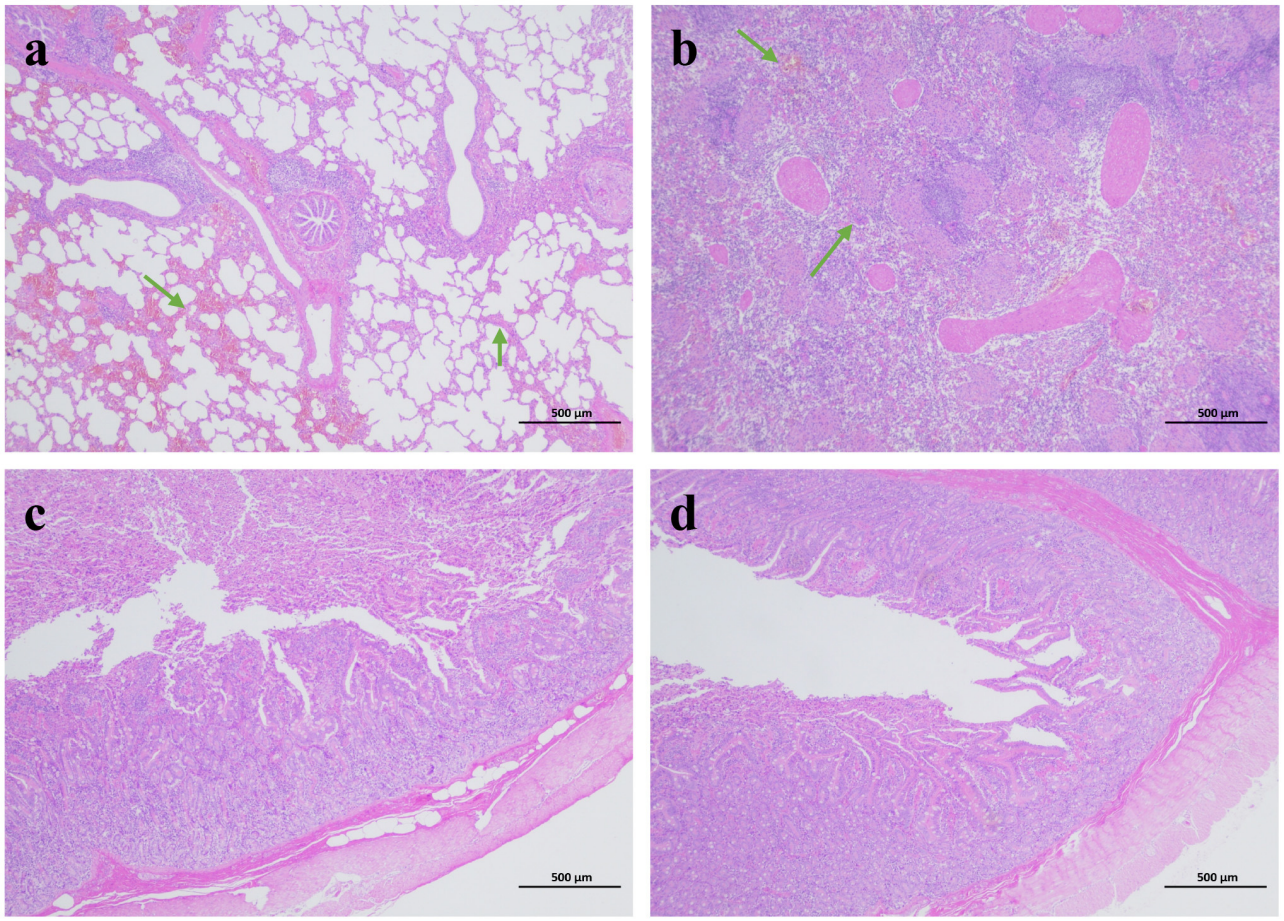
Histopathological analysis of tissues from diarrhea piglets infected with GETV (HE staining). **(a)** Lung. **(b)** Kidney. **(c)** Jejunum. **(d)** Ileum.

Pathological sections of the lungs, kidneys and small intestine with obvious lesions were taken and observed: the alveolar walls were thickened, with increased cellular components and atrophic fusion of alveoli; the main feature of the kidneys was thickening of the renal cortex, and infarcted areas could be observed under the microscope; the villi of the jejunum were generally lowered in height, and villi were atrophied, broken and detached; the villi of the jejunum and the ileum were generally lowered in height, and were severely atrophied. The jejunum and ileum villi were generally reduced in height and severely atrophied (Figure 3).

## 4 Discussion

GETV, a mosquito-borne arbovirus with wide geographical distribution and host adaptability, has been identified as an important animal-borne pathogen because it is widely distributed worldwide and its antibodies/antigens can be detected in a variety of animals (Li et al., 2019; Liu et al., 2023; Rattanatumhi et al., 2022; Takeishi et al., 2022; Wu Q. et al., 2024). However, the epidemiological study of GETV between hosts and vectors is very complex, and more research and data are needed to support it. Before 2006, the pathogen was found in only six provinces in China, but after 2017, the number of provinces infected with the pathogen reached 19, including Heilongjiang, Jilin, Hebei, Shanxi, Shandong, Henan, Gansu, Shaanxi, Sichuan, Yunnan, Anhui, Shanghai, Jiangxi, Hunan, Guizhou, Guangdong, Guangxi, Hainan and Taiwan, with the epidemiological scope expanding year by year (Lan et al., 2024; Li et al., 2019; Lu et al., 2019; Wu Q. et al., 2024; Xing et al., 2020; Yang et al., 2018). Studies over the past 20 years have found that GETV has caused several disease outbreaks in cattle, horses, and blue foxes, and it is generally believed that the pathogen is pathogenic only to cattle, horses, and blue foxes (Liu et al., 2019; Lu et al., 2019; Shi et al., 2019). Since 2017, cases of morbidity and mortality in pig farms in several provinces of China have occurred in clinical piglets infected with GETV, which has resulted in large economic losses in pig farms (Chu et al., 2024; Xing et al., 2020; Yang et al., 2018). To date, there is no specific drug or commercial vaccine avail-able for this disease.

The study of the source of GETV, its ecological distribution in China, its pathogenicity to animals and humans, the determination of its final host, and whether mosquitoes play a vector role between humans and animals are all of great public health significance and animal production application value (Li et al., 2022; Shi et al., 2022b). In this study, we found for the first time that GETV can also cause disease in wild boars, and the symptoms of returning sows to oestrus and abortion, depressed boars with reduced semen quality, diarrhea and death of piglets standing on their hind limbs appeared in the farms where the disease occurred, which were consistent with the pathological changes and clinical symptoms previously observed in domestic pigs (Chu et al., 2024; Pan et al., 2023; Yang et al., 2018). This not only expands the spectrum of infection, but also pro-vides sufficient evidence that GETV can infect wild boar herds and cause clinical disease.

In this study, a total of 97 samples from morbid pig farms were collected and the prevalence of GETV infection was found to be 46.26% (136/77), and these results are consistent with those of other researchers (Liu et al., 2023; Rattanatumhi et al., 2022; Shi et al., 2022a; Wu Q. et al., 2024; Yuen et al., 2022). In general, co-infection is common in morbid pig herds affected by different etiological factors. In recent years, PRoV positivity in Chinese herds has been increasing year by year and has become the most important virus causing swine diarrhea (Zhang et al., 2024). GETV and PRoV were found to be the predominant modes of mixed infection in diarrheal fecal samples, and the co-infection of these two viruses viruses exacerbated the severity of the disease. In this study, the presence of GETV was found for the first time in sow milk and boar semen, so breast milk and semen may also be a mode of transmission of GETV. It was found that inoculation with porcine-derived GETV virus by different routes resulted in more severe clinical symptoms by instillation of GETV than by ocular and intraperitoneal injections, suggesting that different routes of infection influence the pathogenicity of porcine-derived GETV in animals. There is a strong seasonality in the incidence of porcine GETV disease, generally in May-October each year as the high season, mainly outbreaks in hot weather, warm and humid climate of the mosquito active areas, with the increase in temperature, the infection of GETV also showed a rising trend. Animals such as mice, mosquitoes and dogs are storage hosts for GETV, resulting in the virus surviving in the environment for a long period of time and not being easy to remove, leading to an increase in infections and morbidities in livestock such as pigs, and making it correspondingly more difficult to prevent and control the disease (Li et al., 2022; Shi et al., 2022b; Wu Y. et al., 2024). Therefore, continuous monitoring of the epidemiological trend of the disease and molecular biology research on the prevalent strains of the virus will allow for better early prevention of the disease; at the same time, combined with anti-mosquito and anti-mosquito measures, the density of mosquitoes can be lowered to prevent potential vectors from posing a more serious threat.

Comparison of the nucleotide sequence homology of the whole genome of JX2024 with 41 GETV strains available in the Gen Bank database revealed that the sequence was 99.4% similar to that of the JX2024 strain and the Chinese swine-origin strains SD2206/pig/China Shandong/2022 (PP623164), HNNY-1 (MG865966), HNNY-2 (MG865967), and HNPDS-1 (MG865968) were highly similar at 99.4%; however, the lowest similarity was 94.4% with Malaysian mosquito-derived strain MM2021 (AF339484). GETV E2 is a viral outer membrane glycoprotein, and the virus enters into the cell through the interaction of the E2 protein with the receptor of the host cell. It was found that amino acid 253 of the E2 protein is the key amino acid site for the weakening of GETV virulence, and that mutation of this site from lysine to arginine resulted in a decrease in the pathogenicity of GETV in mice. By analyzing the mutation site of E2 protein amino acid sequence, it was found that the structural protein *E2* gene of GETV was highly conserved, and the structural protein encoded by E2 was the antigen recognition site and neutralization site of the virus, which played an important role in adsorption of the target cells, infection of the host, and triggering the body to produce the anti-infective immunity, and the amino acid site of the JX2024 strain was the same as that of Chinese swine SD2206/pig/China_2022 [China swine SD2206/pig/China_2022 (China Swine SD2206/pig/Chinese_2022)]. Shan-dong/2022 (PP623164), GDQY2022 (ON987235), GETV-YL (OL352731), HNJZ-S1 (KY363862), HNJZ-S2 (KY363863), HNNY-1 (MG865966), HNNY-2 (MG865967), HNPDS-1 (MG865968), HNPDS-2 (MG865969), Chinese mosquito source YN0540 (EU015063), Mongolian mosquito source LEIV_17741_MPR (EF631999), Japanese swine source 15-I-1105 (LC212973) and Japanese mosquito source 16-I-676 (LC223132), and MI-110-C2 (LC079086) strains were highly conserved, with amino acid homology above 99.3%. The amino acid stability is more favorable for cross-protection on the vaccine developed based on E2 protein (Miao et al., 2024). Genetic evolutionary analysis of the whole genome nucleotide sequence and the amino acid sequence of the E2 protein showed that the JX2014 strain formed four evolutionary branches, GI, GII, GIII, and GIV, with the 41 strains of Getah virus, which was consistent with the results of the Getah virus clustering reported by Shi et al. (2022b). Further analyses revealed that the JX2024 strain isolated from wild boar in this study was closely related to the Chinese swine sources HNJZ-S1 (KY363862) and HNNY-2 (MG865967) and the Chinese mosquito sources GD2202/mosquito/China/2/2022 (OP747412) and JL17/08 (MG869691). The most recent of these animals are in the same evolutionary branch. The absence of significant vector or host specificity of GETV isolates from these animals and the similar molecular features of these isolates in terms of viral structural genes and molecular genetic evolution of structural genes suggests that domestic pigs, like mosquitoes, may be an infectious source of GETV, and therefore, studies on this virus and its vectors need to be intensified.

Clinical signs, gross lesions and histopathological changes in pigs infected with GETV alone were also evaluated. Moderate to severe atrophic villi were observed microscopically in the duodenum, jejunum and ileum. Serological investigation of Geta virus revealed that antibodies to GETV were detected in serum samples from chickens, ducks, dairy cows, pigs, and beef cattle, suggesting that GETV host animals are widespread and that the mechanism of vector-borne transmission resulting in infections among multiple animals is unknown (Li et al., 2022; Shi et al., 2022b). Our study provides evidence that GETV can cause lethal disease in wild boars. Investigating the routes of transmission of GETV in animals, Investigating the routes of transmission of GETV in animals may help prevent outbreaks of GETV disease in China. Despite the fact that GETV has been in the limelight for more than 70 years, the extent of its harm and impact is not yet clear, and further studies on the biological relevance of genetic variation in the GETV genome to viral pathogenicity, histophilicity, and the mechanism of cross-species transmission are still needed.

## 5 Conclusions

This study showed that GETV can naturally infect wild boar herds and that infected neonatal piglets mainly showed fever, arthritis and death, reproductive disorders in gestating sows, and decreased semen quality in boars. Notably, in this study, the presence of GETV was detected for the first time in sow milk and boar semen. We determined the whole genome sequence of strain JX2024 in aborted fetuses of wild boars from Jiangxi Province, China, and performed pathological observations on visceral and intestinal tissues of piglets with diarrheal. Compared with uninfected piglets, newborn piglets infected with GETV showed inflammatory cell infiltration in the lungs, thickening of the renal cortex in the kidneys, and moderate to severe atrophic villi in the jejunum and ileum. This study fills the gaps in molecular epidemiology, viral genetics, pathogenicity and pathogenesis of GETV in wild boars, and provides a basis for further understanding of the spread of GETV in China.

## Data Availability

The datasets presented in this study can be found in online repositories. The names of the repository/repositories and accession number(s) can be found in the article/Supplementary material.
