# Supplementary material for: First report on identification and genetic characterization of Getah virus in wild boar in China
Source: Front Microbiol. 2025 Mar 24;16:1583023. doi: 10.3389/fmicb.2025.1583023 (PMC11995736; doi:10.3389/fmicb.2025.1583023)
Supplement: Supplementary file 1 [file Table_1.docx]

Table S1. Primers used in this study

| Primer name | Sequence (5'-3') | Target gene (Virus) | Product size |
| --- | --- | --- | --- |
| GETV F | CCAACTCAAACCTTTTACGGAC | Cap (GETV) | 636 bp |
| GETV R | TTTACCTGCGCCTGTCGGGA |  |  |
| PEDV MF | GTATTGGTGGTGAGCGGAAT | ORF1 (PEDV) | 486 bp |
| PEDV MR | CCTGTTCCGCCATTCTATCA |  |  |
| PDCoV NF | CCAAACGCAACCCCAACAATCC | Nucleocapsid (PDCoV) | 329 bp |
| PDCoV NR | CTTCTCAGTGTCTGCAGAGCCG |  |  |
| TGEV SF | TATTTGTGGTTTTGGTTATAATGC | S (TGEV) | 870 bp |
| TGEV SR | GGCTGTTTGGTAACTAATTTGCCA |  |  |
| PoRV F | TATTCAAATATAAGTGATTTAATTCAAC | VP6 (PRoV) | 298 bp |
| PoRV R | TAATACCTGACAGCTTTCTTAATGC |  |  |
| SADS-CoV F | ACACCCAAACCAAGAAGCAG | Nucleocapsid (SADS-CoV) | 497 bp |
| SADS-CoV R | TCCACCATCTCAACCTCYTC |  |  |
| PCV2 F | CGAGAAAGCGAAAGGAACAGAT | Rep (PCV2) | 364 bp |
| PCV2 R | ATCCCACCACTTGTTTCTAGGT |  |  |
| PCV3 F | TTACTTAGAGAACGGACTTGTAACG | Cap (PCV3) | 651 bp |
| PCV3 R | AAATGAGACACAGAGCTATATTCAG |  |  |
| CSFV F | GACACAAGYGCAGGCAAYAG | NS5B (CSFV) | 449 bp |
| CSFV R | AGTGGGTTCCAGGARTACAT |  |  |
| PRRSV | ATGTTGGGGAAGTGCTTGACCGCGT | GP5 (PRRSV) | 603 bp |
| PRRSV | CTAGAGACGACCCCATTGCTCCGCT |  |  |
| JEV F | GTGTGAACTTCTTGGCTTAG | C/pr M (JEV) | 964 bp |
| JEV R | TGTAAGCCGGAGCGACCAA |  |  |
| PPV F | TTACAGAATCAGCAACCTCAC | VP2 (PPV) | 445 bp |
| PPV R | TGGTCTCCTTCTGTGGTAGG |  |  |
| ASFV F | AGTTATGGGAAACCCGACCC | B646L (ASFV) | 257 bp |
| ASFV R | CCCTGAATCGGAGCATCCT |  |  |
